# Urology in ancient India

**DOI:** 10.4103/0970-1591.30253

**Published:** 2007

**Authors:** Sakti Das

**Affiliations:** University of California Davis School of Medicine, USA

**Keywords:** History, India, urology

## Abstract

The practice of medical and surgical measures in the management of urological ailments prevailed in ancient India from the Vedic era around 3000 BC. Subsequently in the Samhita period, the two stalwarts - Charaka in medicine and Susruta in surgery elevated the art of medicine in India to unprecedented heights. Their elaboration of the etiopathological hypothesis and the medical and surgical treatments of various urological disorders of unparalleled ingenuity still remain valid to some extent in our contemporary understanding. The new generation of accomplished Indian urologists should humbly venerate the legacy of the illustrious pioneers in urology of our motherland.

Centuries of our subjugated mindset in pursuit of occidental culture, science and medicine has made it difficult for us to explore and appreciate the original contributions of the ancient pioneers of our own motherland in these particular fields. This paper is a humble attempt to unravel the evidences of the mature albeit sometimes erroneous teachings and practice of urological therapies that prevailed in India a millennium before Hippocrates and about two millennia prior to the era of European stalwarts like Celsius and Galen.

Distinct realms of organized civilization appeared almost simultaneously in the early part of the 3^rd^ millennium BC, along the river valleys of the Euphrates, the Nile and the Indus. Archeological excavations in Harappa and Mahenjodaro in West Punjab, Pakistan, revealed ample evidences of the urban culture of the Indus valley civilization in ancient India.[1] The horse-riding pastoral Indo-Europeans or Aryans from central Asia arrived, settled and merged into this civilization. They spoke a variant of early Sanskrit, now called the Vedic Sanskrit.[2]

Medical doctrines are first encountered in the religious texts of that period called the Vedas compiled in successive generations from 3000 to 1000 BC.[3] In chronological order, the four Vedas namely the Rig, Yajur, Sam and Atharva Veda chronicle the Vedic hymns as oral religious literature that are still recited during weddings, funerals and other socio-religious occasions in contemporary India.

The seminal reference to urologic ailments in human history is encountered in the Atharva Veda dealing with urinary retention. It specifies the management with camphor and indigenous herbs to be anointed on the abdomen along with chanting of the appropriate hymns. The Atharva Veda contains a number of recommendations for the alleviation of specific physical ailments including sexual dysfunction. The fourth canto of Chapter 4 narrates the hymns to be recited and herbal roots to be ingested with boiled milk for the purpose of enhancing sexual vigor. In the 10^th^ canto of Chapter 6, there are explicit advices about amulets blessed by mantras to improve male tumescence. Aphrodisiac potions of medicinal herbs and clarified butter are to be embalmed to excite and entice the female. Chanting of specific mantras or hymns were integral parts of most therapies.[4]

The Vedic hymns mostly dealt with religious rituals. The Vedic era was soon juxtaposed by the periods of Brahmanas and Upanishads. The Brhamanas are essentially appendices to the Vedas. The later mystical writings of the Upanishads compiled around 800 BC were devoted to philosophic discourses on the transcendental manifestations of the supreme creator and his universe. This large body of about 108 Upanishads contained teachings of esoteric doctrines of asceticism, discussions about Brahma the supreme entity, Atman the self or soul and discourses on ethics etc.[5]

Sexuality and information about sexual congress appear sporadically in several Upanishads. The productive process of the divine was thought of in terms of sexual union. The details about the physiology of gestation and embryogenesis are amazing. In the sixth chapter of the Brihadaranyak Upanishad, several cantos mention the proper methods of initiating foreplay, enticing the female to coitus and coital methods for the purpose of pleasure and procreation with special attention to the sexual fulfillment of the female partner. The 13^th^ chapter of the Chhandogya Upanishad details the coital process and rationalization of sexual congress for the propagation of the family.[6]

Around 9^th^ century BC, all over the ancient universe, separate centers of civilization in Mesopotamia, Greece, China and India were revealing signs of new evolution of ethics, conscience and rational thinking that challenged the prevailing religions of custom and magic. In this era of so called Brahmana literature also known as the Samhita period, the art of medical practice in India reached its zenith. The two stalwarts in this period are Charaka in medicine and Susruta in surgery.

The anthology of Charaka called the *Charaka Samhita* details ayurvedic medicine in six elaborate volumes. The main emphasis of Charaka was on the maintenance of a healthy disease-free ambience in life by achieving a balance of the three primary functional elements - Vayu (air), Pitta (bile) and Kafa (phlegm). Of course, the implications of these elements go beyond their literary translation of air, bile and phlegm. Each of these terms acquires variable connotations depending on the context of their use. For example, Vayu comprehends all phenomena of motion in life – the cell development, circulation, nervous system etc. Pitta or bile stands for metabolism and Kafa or phlegm implies heat regulation and glandular secretory activities. Bodily dysfunctions or diseases are caused by an imbalance of these primary elements. Ayurvedic medicine attempts to correct and restore the balance.

*Charaka Samhita* contains several sections on urologic ailments.[7] The entire fourth chapter of volume 2 is devoted to urinalysis and clinical interpretations based upon the color, consistency, turbidity, stickiness, presence of blood, semen, pus and fat in urine. Charaka analyzed the urinary findings with the symptoms of frequency, dysuria, polyuria, intermittency, fever, malaise, nausea etc to arrive at an etiopathological explanation of the individual ailments. Later in the same volume he discussed urinary retention precipitated by dietary and alcoholic indiscretions.

In volume 3, chapter 2, there is a long discussion on sexuality and erectile dysfunction. Twelve varieties of aphrodisiacs are categorized by age and specific complaints of quality of erection, maintenance of erection and premature ejaculation. Charaka advised abstinence until age 16 and after age 70.

Another chapter is devoted to febrile symptoms. Charaka prognosticates that high fever associated with painful seminal discharge is almost incurable and often fatal. One wonders if he was observing patients with sepsis from acute prostatitis. In two chapter, Charaka describes urethral and bladder instillations for certain calculus diseases and for cystitis in women.

In volume 5, chapter 26, Charaka mentions symptoms of frequency, strangury, hematuria and occasional urinary obstruction from vesical calculus. He mentions the shape and surface characteristics of various calculi and offers his theories on the etiology. Various herbal medications are recommended for oral intake as well as to be anointed on the abdomen. In recalcitrant situations he advised referral for surgical intervention.

Somewhat contemporary to Charaka was the great Indian sage surgeon Susruta. In his historical chronicle, Whipple stated - “All in all, Susruta must be considered the greatest surgeon of the premedieval period”.[8] The exact chronology of Susruta is hard to speculate, but most western Indologists have placed him between 600 to 1000 BC.[9–11]

Susruta, a savant surgeon and philosopher, but above all a great teacher, compiled a monumental treatise in surgery, the *Susruta Samhita* which is the earliest oral rendition of systematic teaching of surgery. It was first translated into Arabic in the 8^th^ century and later into Latin by Hessler, into German by Vellurs and into English by Hoernle.[12] More recently, Kaviraj Bhishagharatna has published an English version in 1910 with a later edition in 1963.[13]

Susruta indexed five principal sections in his text - 1) Sutrasthana or primary principles with 46 chapters dealing with basic principles of medical sciences and pharmacology, 2) Nidana - 16 chapters dealing with pathological concepts, 3) Sarirasthana - 10 chapters on human anatomy, 4) Chikitsasthanam - 34 chapters on medical and surgical managements and 5) Kalpasthanam - eight chapters on toxicology.

The concept of anatomy in the *Samhita* is certainly inadequate from our modern perspective, but we must applaud the painstaking perseverance of Susruta in trying to learn anatomy by allowing the corpse to decompose in the river and then scrubbing layer by layer to decipher the structural details. His text deals with osteology, myology, splanchnology and embryology. Although the inadequacy and inaccuracy of his concepts are evident now, we must agree with Hoernle who studied and translated the *Samhita* and stated about Susruta's anatomic writings. “Its extent and accuracy are surprising when we allow for their early age and their peculiar method of definition”.[12]

Susruta provided minute details of the manufacture and maintenance of at least 125 surgical instruments including 28 varieties of catheters, sounds and irrigating syringes [Figure 1]. He gave precise measurements, recommended the metal too be used and advised on cleansing with alkalies and caustics[14]

**Figure 1:**
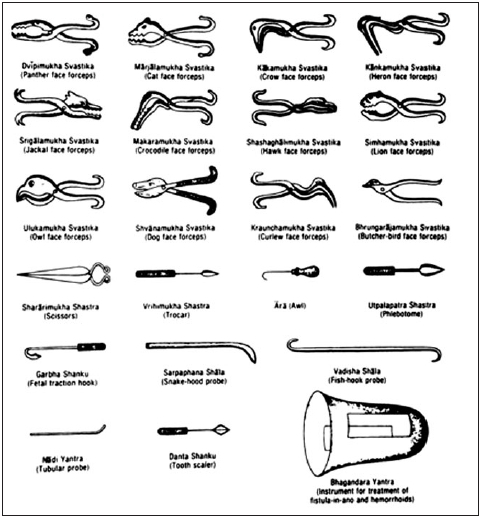
Several surgical instruments as described by Susruta. Redrawn from Mukhopadhayaya G[14]

The pivotal highlight of Susruta's surgical repertoire was the surgery of nasal reconstruction or rhinoplasty [Figure 2]. His technique was later revived by the Italian surgeon Tagliacozzi[15] and the first description in English appeared in The Gentleman's Magazine in 1794.[16] Ackernecht has aptly observed -“There is little doubt that plastic surgery in Europe which flourished in medieval Italy is a direct descendant of classical Indian surgery”.[17] Even today plastic surgeons refer to the pedicled forehead flap as the Indian flap. Susruta Samhita contains descriptions of laparotomy, repair of intestinal injuries as well as numerous other surgeries for hernia, hydrocele, anal fistula, fractures, amputations, cataract couching etc to mention a few.

**Figure 2:**
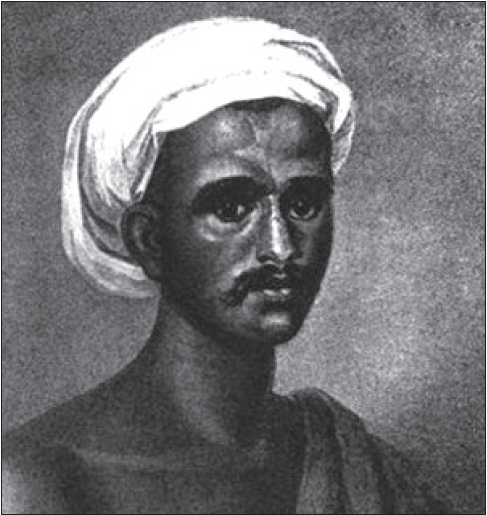
Engraving about rhinoplasty by Susruta that appeared in The Gentleman's Magazine, October 1794

Susruta discussed various urological ailments with conjectures about their pathogenesis followed by detailed management. Several chapters deal with urinary tract infection in both genders. He mentioned a number of urethral probes, dilators and irrigating syringes for instillation of medications.

His detailed management of urethral stricture is quite striking - “In a case of Niruddhaprakasha (stricture of the urethra), a tube open at both ends made of iron, wood or shellac should be lubricated with clarified butter and gently introduced into the urethra. Thicker and thicker tubes should be duly introduced every 3^rd^ day. The urethra passage should be made to dilate in this manner and emollient food should be given to the patient. As an alternative, an incision should be made into the lower part of the penis avoiding the sevani (raphe) and it should be treated as an incidental ulcer”. The causes and prevalence of urethral stricture have changed little with time. It is amazing that the principles of its management with dilatation and urethroplasty that Susruta proposed three millenia ago still remain valid today.[18]

Susruta devoted a whole chapter on penile sores. Depending on their clinical characteristics, specific treatments were recommended that included scarification, coring, excision of condylomatous growths, local astringents and application of leeches.

An elaborate chapter in the second volume of the Samhita deals with sexual hygiene with specific admonitions against certain sexual behaviors. The chapter ends thus - “Hence a healthy and passionate man possessed of the necessary fecundating elements under the course of proper Vaji Karana remedy should cheerfully go unto and enjoy the pleasures of the company of a woman beautiful in looks, modest, virtuous and equally passionate”.

In a subsequent chapter he deals with erectile dysfunction; mentions etiologies like congenital, psychogenic organic impotence and premature ejaculation. The treatments include Vaji Karana defined as remedies that “make a man sexually strong as a horse (Vaji) and enables him to cheerfully satisfy the heat and amorous ardors of young maidens”. His treatment regimen takes a holistic approach with attention to soothing erotic ambience, music, diet and various exotic aphrodisiacs.

Susruta provided the most fascinating details about urinary calculus disease. He described several varieties of urinary calculi, their clinical manifestations and emphasized dietary indiscretion as the main etiological factor.

In chapter 7 of Chikitsasthanam he mentions initial medical management with diet, fluids, alkali and bladder instillations. This is followed by critical narration of his pioneering surgery of perineal vesicolithotomy. The present author has elaborated this in a separate publication.[19] Susruta also narrated the lithotomy technique in females. Postoperative measures included regular fomentation in warm bath, diuretics and urethral irrigation with a Vasti Yantra (bladder syringe). In case of continued postoperative leakage, Susruta suggested - “The wound should be cauterized with fire in the event of urine not flowing through its natural passage after the lapse of seven days”.

After describing the surgical anatomy and complications that may arise from vesicolithotomy, Susruta concluded - “The surgeon who is not well cognizant of the nature and position of the vulnerable parts in the 8 srotas (ducts) namely the perineal raphe, spermatic cords, ducts of the testes, Yoni (vagina), the rectum, the urethra, urine carrying ducts or ureters and the urinary bladder and is not practiced in the art of surgery, brings about the death of many innocent victims”.

In the western civilization, Hippocrates (460-370 BC) mentioned calculus disease and cautioned against the misadventures of surgical lithotomy by charlatans. Such admonition was quite justified considering the lack of systematic surgical teaching and practice beyond management of wounds and fractures. Much later Ammonius (283-247 BC) and Celsus (1^st^ century AD) provided technical details of perineal vesicolithotomy that closely resembled what Susruta had described. Ammonius and Celsus advocated a transverse crescent-like incision in the mid-perineum[20] followed by a vertical incision over the calculus at the bladder neck, whereas Susruta several centuries earlier approached through a vertical incision just lateral to the perineal raphe.

The socioeconomic and cultural exchanges between India and the GrecoRoman domain were prevalent in ancient times and became more established after the invasion of India by Alexander the Great in 326 BC. These exchanges are well chronicled by historians like Strabo, Pliny and Plutarch. It is conceivable that in the course of the cultural exchange between the two civilizations, the earlier technique of vesicolithotomy of Susruta permeated into Hellenic medicine as did his concepts of medical ethics, anatomy, physiology and treatment of diseases.

Medicine in ancient India certainly had spread its influence on Arabic medicine. There are striking similarities between the surgical instruments depicted as wall sculpture in the temple of Kom Ombo in Egypt and those described by Susruta. Outside India the first translation of Susruta Samhita appeared in Arabic as Kitab-i-Susrud by Abil Saibial in the 8^th^ century AD. Sir William Hunter stated – “Arabic medicine was founded on translations from Sanskrit treatises and in turn European medicine down to the 7^th^ century was based on the Latin versions of those Arabic translations of Hindu medicine”.[21]

Needless to mention that Ayurvedic treatment of urologic ailments prevailed in India for many centuries with distinct contributions of other stalwarts not just limited to the encyclopedic Kamasutra by Vatsayana in 5^th^ century AD.[22] However, the influence of Islamic medical practices during the Mughal era ushered in its gradual decline followed by the overwhelming ascendancy of Western medicine during the era of the imperial British colonialism.

Urology in modern India has made emphatic strides and has established itself as a significant tour de force in the global urology arena. In that monumental progress we need to humbly remember the legacy of our illustrious pioneers in urology in our motherland whose ancient urologic practice and teaching of unparalleled ingenuity still remain valid in principle in the contemporary context.
